# Nanotechnology Approaches for Enhanced CNS Drug Delivery in the Management of Schizophrenia

**DOI:** 10.34172/apb.2022.052

**Published:** 2021-10-02

**Authors:** Rajalakshmi Rajendran, Krishnakumar Neelakandha Menon, Sreeja Chandrasekharan Nair

**Affiliations:** ^1^Amrita School of Pharmacy, Amrita Vishwa Vidyapeetham, Kochi-682041, Kerala, India.; ^2^Amrita Centre for Nanosciences and Molecular Medicine, Amrita Institute of Medical Science and Research Centre, Amrita Vishwa Vidyapeetham, Kochi-682041, Kerala, India.

**Keywords:** Schizophrenia, Blood-brain barrier, Biomarkers, Drug delivery systems, Nanotechnology

## Abstract

Schizophrenia is a neuropsychiatric disorder mainly affecting the central nervous system (CNS), presented with auditory and visual hallucinations, delusion and withdrawal from society. Abnormal dopamine levels mainly characterise the disease; various theories of neurotransmitters explain the pathophysiology of the disease. The current therapeutic approach deals with the systemic administration of drugs other than the enteral route, altering the neurotransmitter levels within the brain and providing symptomatic relief. Fluid biomarkers help in the early detection of the disease, which would improve the therapeutic efficacy. However, the major challenge faced in CNS drug delivery is the blood-brain barrier (BBB). Nanotherapeutic approaches may overcome these limitations, which will improve safety, efficacy, and targeted drug delivery. This review article addresses the main challenges faced in CNS drug delivery and the significance of current therapeutic strategies and nanotherapeutic approaches for a better understanding and enhanced drug delivery to the brain, which improve the quality of life of schizophrenia patients.

## Introduction

Schizophrenia is a severe mental disorder characterised by auditory and visual hallucinations, bizarre thoughts, social withdrawal and incoherent thoughts. It is a disorder related to brain volume and can be considered a progressive brain disease, worsening over the years. According to the World Health Organization (WHO), about 20 million people are affected with schizophrenia worldwide.^1^ People with schizophrenia die at an early life 3-4 times more likely than the general population. The prevalence of schizophrenia varies globally, although most cases are reported from the Middle East, East Asia and the United States.^2^ The disease develops due to the change in the two crucial neurotransmitter levels in the brain, dopamine and serotonin. Genetic factors such as deletion in chromosome 22 constitute a small percentage of cases for schizophrenia.^3^ Environmental factors and lifestyle modifications are also causes for the disease. The disease’s signs and symptoms include hallucination, delusion, disordered thinking, and cognitive impairment with a high risk of suicidal behaviour. The peak age for the onset of the disease is 20-35 years and is more frequently diagnosed in males than in females.^4^

As the disease progresses over the years, new therapeutic strategies should be implemented for the management. Current therapeutic strategies mainly rely upon the neurotransmitter hypothesis for the development of drugs. These approaches include the dopaminergic hypothesis, serotoninergic hypothesis, glutamate hypothesis, and cholinergic hypothesis. Above all, the dopaminergic hypothesis is the primary strategy. It is based on a fundamental reason for the development of schizophrenia is increased dopamine level in the region of the nucleus accumbens of the brain.

Furthermore, decreased dopamine levels in the frontotemporal region of the brain finally lead to positive and negative symptoms of the disease, respectively.^5^ The treatment methods aim at reducing dopamine levels and thereby resolving the hyperactivity. On the other hand, the serotoninergic hypothesis is based on the fact that serotonin receptors are more concentrated in raphe nuclei, a region involved in mood control and cognition. In schizophrenia, 5HT-2A receptor blockade is a crucial mechanism of action of atypical antipsychotics. Besides, glutamate receptor blockade also plays a pivotal role in antagonising the actions of other neurotransmitters and exhibits symptoms in schizophrenia. The factors that trigger the disease’s risk include family history, exposure to viruses, toxins or malnutrition while in the womb, particularly during first and second trimesters, stressful life circumstances, older paternal age, and taking psychoactive drugs during adolescents young adulthood.^6^ It is suggested that rehabilitation, psychosocial support, cognitive therapy, and other lifestyle modifications improve the patient’s quality of life to a great extent other than pharmacological treatment. Early diagnosis of the disease helps to improve the life of the patient. Novel nanotherapeutic approaches play an essential role in managing schizophrenia through selectivity, specificity, safety and targeted drug delivery. This review addresses the current prevalent hypothesis on schizophrenia, the concept of fluid biomarkers as a diagnostic agent and various drugs and drug delivery approaches in managing schizophrenia. We also issue the various alternative nanotherapeutic approaches with their merits and limitations.

## Current therapeutic strategies and future perspectives in schizophrenia

### Dopaminergic hypothesis

The dopamine hypothesis is one of the most recognised theories for the present investigators in schizophrenia research. As mentioned earlier, this hypothesis hyperlinks to the primary aetiology of schizophrenia. The hyperactivity of dopamine in the nucleus accumbens, and the hypoactivity of dopamine in the frontotemporal region results in progressive psychotic episodes.^7^ Increase in the dopamine level due to hyperactivity of the ventral hippocampus demonstrated in a rat model.^8^ Revised theories on dopamine by Davis et al state that dopamine in the mesolimbic area and dopamine’s hypoactivity in dopamine the prefrontal cortex results in schizophrenia symptoms.^9^ Amygdala and prefrontal cortex are involved in cognition wherein patients with schizophrenia; dopamine is dysregulated, making the patient weak in emotional processing.

**Figure 1:**
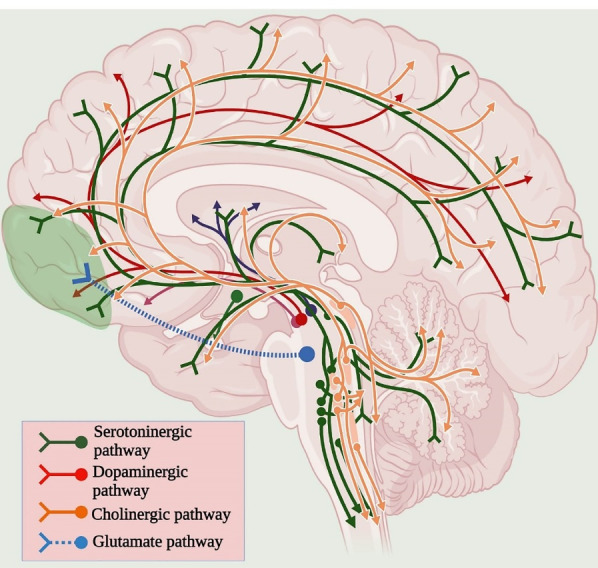
Neurotransmitter pathways associated with Schizophrenia.

Antipsychotics based on dopamine hypothesis is usually followed in patients with schizophrenia to normalise the dopaminergic activity by providing antidopaminergic drugs, specifically dopamine D2 receptor antagonist. Most antipsychotic drugs block the D2 receptor in the mesolimbic pathway to treat psychotic symptoms. However, it is not sufficient to treat all the symptoms of schizophrenia; in addition, the extension of the dopamine hypothesis is required to establish the role of other neurotransmitters involved in schizophrenia. Various neurotransmitter pathways involved in the pathophysiology of schizophrenia is indicated in Figure 1.^10^

#### Dopamine receptor antagonists and stabilisers

The majority of the antipsychotics are developed based on the dopamine hypothesis to antagonise dopamine by competitively binding to its receptor or stabilising its effect rather than blocking the receptor, such as currently used aripiprazole.^11^ Another antipsychotic drug (−)-OSU6162, recently accepted by FDA, is a dopamine stabiliser that elicits a dose-dependent activity and inhibits D2 receptor selectively, thus reduces peripheral antipsychotic side-effects.^12^ However, the currently available antipsychotic drug, pimavanserin, used in treating Parkinson’s disease, is an exception.^13^ Other than dopamine, treatment approaches focusing on other neurotransmitters may avoid medication non-adherence, which is a frequent problem facing in the treatment of schizophrenia.

### Glutamate theory

Glutamate is an excitatory neurotransmitter found abundantly in the brain. N-methyl-D-aspartate receptors (NMDARs) mediate the glutamate neurotransmission connected to the cortex, limbic system, and thalamus regions of the brain in schizophrenia. The relationship between glutamate and schizophrenia is observed when reduced glutamate levels were observed in the cerebrospinal fluid of schizophrenia patients.^14^ The patients on drugs such as Phencyclidine and ketamine show psychotic episodes since they are NMDAR antagonist. Contrarily patients on NMDAR agonists show their required therapeutic outcome.^15^ Therefore, drugs focused on glutamate neurotransmission are a hopeful target because of their vital role in schizophrenia regarding intellectual disabilities and negative psychotic symptoms.

#### Glutamatergic agonists

Glutamate, the most abundantly seen neurotransmitter, is a vital target involved in the pathophysiology of schizophrenia. Other than dopamine antagonist, NMDAR agonists gain broad recognition as an effective substrate in schizophrenia. NMDAR agonists, when used directly, causes neurotoxicity, and therefore indirect agents are used, for example, glycinergic agents such as serine and cycloserine.^16^ In addition, glycine transport inhibitors are also extensively studied, such as bitopertin.^17^ In preclinical studies, these agents show increased glycine bioavailability since they act as co-agonist in NMDAR sites. These agents portray moderate benefits by alleviating negative symptoms in schizophrenia.

In animal studies, Metabotropic receptor-1 blockers acted as the target for alleviating positive symptoms in schizophrenia.^18^ In addition, Metabotropic receptor-2 and 3 agonists such as pomaglumetad methionil reduce glutamate availability at the site of action.^19^ These agents show promising results in the early stages of schizophrenia; further analysis should be done by clinical trials to ensure therapeutic safety and efficacy.

### Serotoninergic hypothesis

Serotonin, chemically 5-hydroxytryptamine (5-HT), is a neurotransmitter studied along with dopamine in the pathophysiology of schizophrenia. It was based on the fact that serotonin receptors mediate the indoleamine (say, LSD) and phenethylamine (say, mescaline) activity in the brain’s locus coeruleus and cerebral cortex regions enhances glutamate neurotransmission.^20^ Risperidone and clozapine are drugs known to have dopamine- serotonin antagonising effect, an exciting area to study as a better drug target. However, authentication for the role of serotonin abnormality is not yet available, though serotonin receptors are the target of interest in the treatment of schizophrenia.

#### Serotonin agonists

Except for clozapine, all other second-generation antipsychotics do not benefit from treating schizophrenia other than providing relief from extrapyramidal side effects. Various strategies were under investigation, such as 5-HT1A receptor agonists, Serotonin reuptake inhibitors, agonist and antagonist of 5-HT2C, the antagonist of 5-HT3 and 5-HT6.^21^ These agents are investigated alone or in combination with dopamine antagonists. 5-HT2A/D2 antagonist and 5-HT partial agonist are available in the market along with further research. Presently, a 5-HT3 receptor antagonist such as Ondansetron is now chosen as an adjunctive agent due to its potential anti-inflammatory action, which will help to overcome the negative and positive symptoms of schizophrenia.^22^ Ondansetron as adjunctive therapy is now under phase III clinical trial, evaluating for negative symptoms of the disease.^23^ Another antipsychotic drug Lurasidone, is recently introduced for its 5-HT7 antagonistic activity, which is benefitted in psychotic mood swings.^24^

### Cholinergic hypothesis

Smoking is an under-defined risk factor for schizophrenia observed in the majority of patients.^25^ The rate of smoking proportionally increases the severity of the ailment. Patients reported that the reason for them to smoke is to overcome the negative symptoms and compensate for the medicine’s undesirable effects. These observations explain the effort of the patient to reduce the inadequacy of nicotinic cholinergic receptors.^26^

The defective sensory functioning in patients with schizophrenia results from weak P-50 dynamic responses to repetitive auditory stimuli.^27^ Desensitisation of the nicotinic receptor occurs due to smoking, which results in attenuation of impaired auditory functioning, which is associated with the α-7 gene of the nicotinic receptor.^28^ Therefore, cholinergic agents such as α-7 nicotinic receptor agonist enhance the treatment’s efficacy by alleviating specific symptoms in schizophrenia. Nicotinic-cholinergic receptor-mediated agonist is a clear focus in schizophrenia.

#### Cholinergic agents

Various studies have focused on cholinergic neurotransmission because of its involvement in the pathophysiology of schizophrenia. The α-7 nicotinic receptor agonists such as EVP-6124, now in phase-III trials and many other agents, including GTS-21 and AQW051, are under early developmental stages.^29^ These agents are targeted for cognitive impairment associated with schizophrenia, but undesirable effects and pharmacokinetic profile are hindering factor for developing an effective cholinergic agent.

### γ-Aminobutyric acid (GABA)

Gamma-aminobutyric acid is a naturally occurring amino acid and an inhibitory neurotransmitter found in the central nervous system (CNS). The role of GABA and dopamine in schizophrenia is reported in certain studies and animal models.^30^ The subset specifically affected in schizophrenia is the chandelier subtype of parvalbumin-positive GABA neurons.^31^

In schizophrenia, there is a decrease in the mRNA coding for GABA producing enzyme GAD67, resulting in decreased neuronal density.^32^ In addition, the levels of GAT-1 mRNA, which reuptakes GABA, is found to be decreased in the brain.^33^ Reduced GAT-1 increases the availability of GABA in the prefrontal cortex. The role of GABA in schizophrenia is not clearly understood, but clinically the administration of GABA agonists as adjunctive therapy improves the therapeutic outcome. Further research is required to establish the role of GABA in schizophrenia and elucidate the relationship between GABA and other neurotransmitters in the treatment of schizophrenia.

#### GABA receptor positive allosteric modulators

GABA is a neurotransmitter mentioned in the pathophysiology of schizophrenia. The GABA receptors have three allosteric sites, out of which one is the benzodiazepine binding site that mediates chloride gated ion channel.^34^ Specific GABA-A agonists and GABA-B antagonists are currently under investigation in the treatment of schizophrenia.^35^

### Inflammation

Other topics of interest in the research of schizophrenia are inflammation and oxidative stress. Altered complementary proteins involved in innate immunity activation such as C1, C3 and C4 are demonstrated in schizophrenia.^36^ These complementary proteins may make use of microglia to interrupt the synapses; as a result, synaptic pruning is accelerated.^37^

Inflammation affects neurotransmitters such as inflammatory cytokines, and neurotransmitter levels in specific regions of the brain have a role in the pathophysiology of schizophrenia. An example for the inflammatory model indicates that enhanced stimulation of inflammatory markers in the pregnancy causes the fetus to be more vulnerable to schizophrenia.^38^ Anti-inflammatory agents’ help in this context which will be discussed later. Another immune model describes gluten sensitivity; that is, it causes high transglutaminase antibodies, and the patients with gluten sensitivity benefit from a gluten-free diet.^39^

#### Anti-inflammatory agents

Anti-inflammatory agents are better targeted in the pathophysiology of schizophrenia since it is involved in the disorder. Commonly used anti-inflammatory agents are cyclooxygenase enzyme inhibitors like celecoxib is administered in patients with exacerbations of schizophrenia as add-on therapy with risperidone.^40^ The results were quite promising that showed much improvement in patients who received celecoxib compared to those who did not receive the same. Another study implicated that the antibiotic minocycline can inhibit microglial activation by crossing the blood-brain barrier (BBB) in patients with schizophrenia and improving cognition.^41^ Monoclonal antibodies are also under investigation to act against the inflammatory cytokines, their action is feasible, but further research has to be done for evaluating the therapeutic outcome.^42^

Various neurotransmitters and immune-mediated responses are connected and found to have a role in the pathophysiology of schizophrenia. Thus treatment methods based on a single neurotransmitter will not give a better therapeutic outcome. Hence, pointing the novel therapeutic approach towards the complex neurotransmitter network is essential for better understanding schizophrenia. It can be achieved by considering a method in computational biology, the graph theory, which uses nodes and edges for having a clear outlook over the model.^43^ In this method, we consider all the above said theories and hypotheses as different nodes connected to a median and any imbalance inside the system would affect the whole set-up.^44^ Similarly, the system may get benefitted when every process within the system is in homeostasis.

## Fluid biomarkers for schizophrenia

Most of the current treatment methods for schizophrenia focus on alleviating the symptoms rather than aiming at the pathophysiology behind the disease. Researches have been done to bring out new agents that can alter the progression of the disease, but none of them has been expressed. The above said difficult situation can be sorted out by introducing biomarkers that can play a vital role in detecting the disease.^45^ According to National Institute of Health (NIH), a biomarker is an objective, quantifiable indicator of a biological process.^46^

**Figure 2:**
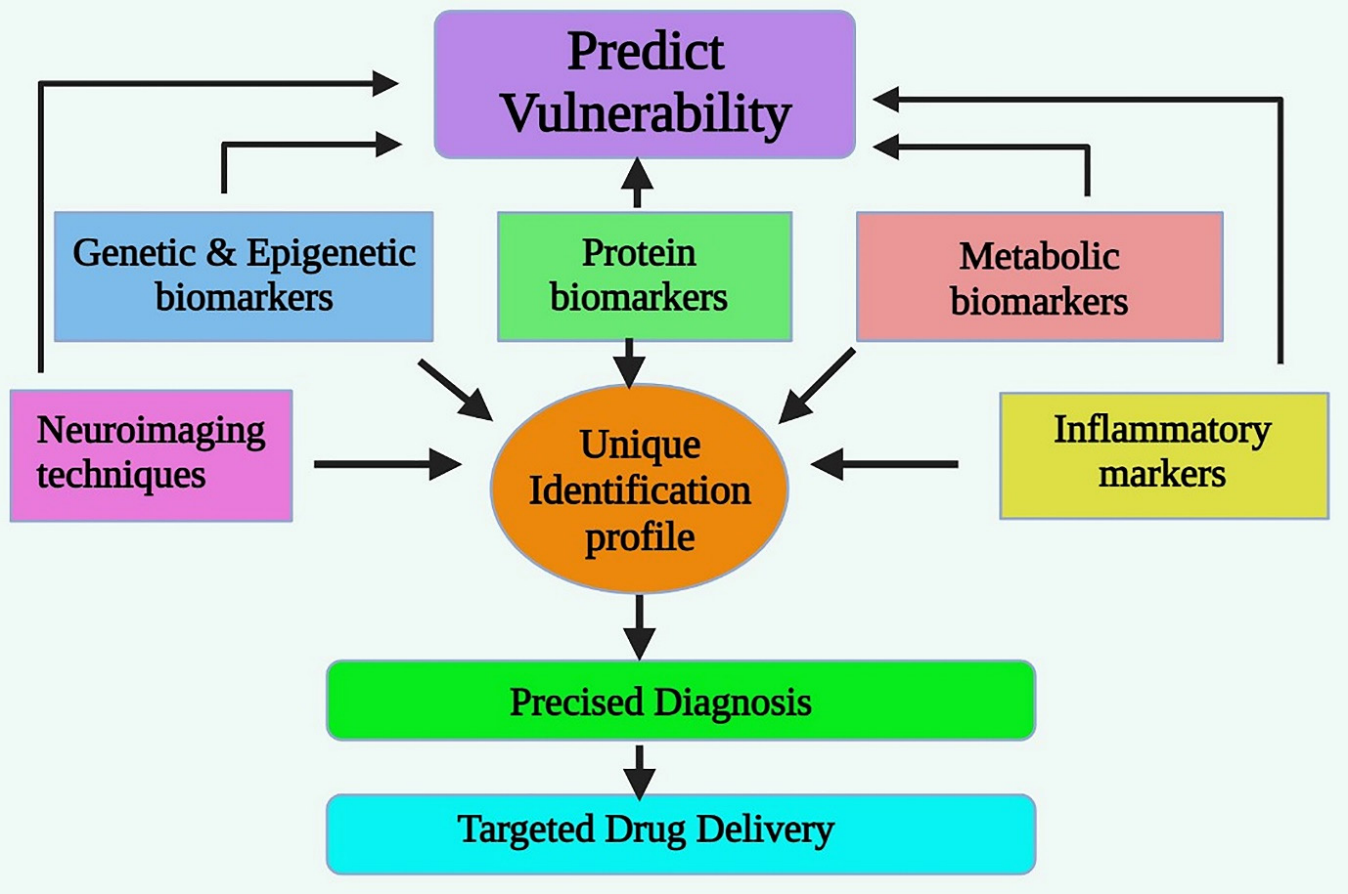
The Potential Biomarkers Clinically Utilized for Schizophrenia.

After screening and filtering the available data on potential biomarkers available for the diagnosis and clinical utility in schizophrenia, there are six types of biomarkers established with potential and inconclusive results and Figure 2 shows diagrammatic illustration of the clinical utility of biomarkers in schizophrenia. Two are from neuroimaging techniques; two are genetic, epigenetic, protein, metabolic and other biomarkers. Brain sampling is difficult to be done on a macroscopic scale, although structural and functional markers are detected. Eventually, the combination of markers can also be done to test the hypothesis and interpret the result.^47^

### Neuroimaging techniques

Neuroimaging plays a significant role in the qualitative analysis of brain structure and functioning. Therefore, neuroimaging is a novel, promising tool for analysing morphological structures and the functioning of brain tissue. In schizophrenia, neuroimaging techniques help in identifying the brain areas associated with cognition, hypo-activation and hyper-activation.^48^ Studies reveal that prefrontal cortical volume is associated with decreased cognitive function, logical thinking, execution and critical approach.^49^ There are two types of neuroimaging studies- structural imaging and functional brain imaging. Structural brain imaging aids in identifying the anatomical changes in brain structure due to schizophrenia. Functional imaging includes magnetic resonance imaging (MRI), magnetic resonance spectroscopy (MRS), also fluorodeoxyglucose-positron emission tomography (FDG PET).^50^ MRI helps identify the individuals with a high risk of schizophrenia with a substantial reduction in grey matter volume in the prefrontal cortex, temporal, parietal and occipital cortices.^51^ Similarly, functional MRI (fMRI) relates the affected individuals’ fluctuations in brain oxygen demand.^52^ One of the limitations hidden in this technique is that the abnormal motif coupled to cognition and cortical volume could also be susceptible to neuroleptic drug effects.^53^

### Genetic biomarkers

Mental disorders are genetically ambiguous, even clinically, diverse, variable, and interrelated.^54^ In many medical and psychological disorders, genetic and gene expression research is becoming more unified for both humans and rodents.^55^ There is still reliable evidence of biological and epigenetic effects on schizophrenia genetic disposition.^56^ The use of blood cells to perform microarray studies has become increasingly popular due to the numerous advantages it provides, including the possibility to collect larger sample sizes with a minimally invasive procedure.^57^ Genomic technology innovations have led to a better understanding of psychiatric disorders, providing information about numerous genes that affect brain development.^58^ The biggest drawback of genetic therapies in schizophrenia is the quantification of operational gene regulation. Since proteins go through multiple variations from transcription to post-translation, and mRNA overload cannot accurately estimate protein levels in regular or high-stress situations, such as in diseases.^59^ Antipsychotic behaviour-related peripheral gene expression studies, like therapeutic response studies, are few; however, they are promising while pointing to the need for more research.^60^

### Epigenetic biomarkers

All fundamental psychiatric syndromes are complex, heterogeneous conditions because of the interaction of numerous factors, including genetic, neurobiological, cultural, and lifestyles background. Epigenetic mechanisms deal with the immensely complex molecule, the DNA in the cell nucleus, and many forms of histone and DNA changes and alterations in many styles of non-histone proteins non-coding RNAs.^61^ Only some studies of non-coding RNA were carried out for biomarkers in human subjects; even as in preclinical investigations, there was an explosion of non-coding RNA research.^62^ A vicinity of studies associated with non-coding RNA and gene expression is microRNA (miRNA), which could control the extent and translation of mRNA.^63^ Schizophrenia is characterised by using gene expression changes inside the cerebral cortex and other brain regions. Because transcriptional mechanisms rely upon active chromatin remodelling, Dysregulated gene expression in the schizophrenic brain would be expected to give epigenetic alterations to their regulatory regions.^64^ DNA methylation happens with the addition of a methyl group to the C5 position of cytosine (5’-met C), predominantly at cytosine-guanine dyads (CpG websites).^65^ Regarding modifications in DNA (DNA methylation), a few candidate gene promoters are RELN, which encodes reelin, whose promoter indicates accelerated methylation in the prefrontal cortex and positive variant brain regions in human beings with schizophrenia. SOX10, which encodes a transcription thing important in improvement. Human leukocyte antigen (HLA) is noteworthy given the purported role of inflammation in disease pathogenesis and other promoters with an altered metamorphosis in schizophrenia along with brain-derived neurotrophic factor (BDNF), serotonin 1A receptor (5HTR1A), and catechol-O-methyl transferase (COMT).^66^ Another epigenetic alteration in schizophrenia is correlated with epigenetic dysregulation of GAD1 in the prefrontal cortex, including excessive ranges of repressive DNA and histone methylation.^67^ To find out new biomarkers in the epigenetic area of schizophrenia, it is essential to recognise how genetic vulnerabilities interact with a person’s life experience to establish solid adjustments at unique genomic loci, which then manipulate the degrees of gene expression or indelibility.^68^ Limitations of epigenetic biomarkers are correlated with the complexity of schizophrenia; for instance, the methylation values fluctuate among leukocytes subtypes, and methylation styles in whole blood can be divergent from those detected in particular cell types.^69^

### Protein biomarkers

Protein biomarkers have enough potential due to their wide variety and critical involvement in schizophrenia psychopathology. Multi-analytic and array profiling techniques permit the simultaneous detection of hundreds of proteins with high sensitivity and accuracy. They may be efficiently implemented to identify biomarkers (or clusters of biomarkers) that correlate with the disorder.^70^ A proteome contains the whole set of proteins in a biological system (cellular, tissue, or organism) in a specific state at a given time.^71^

Available tissues, such as cerebrospinal fluid, blood serum, plasma, and others, including fibroblasts, the liver, and urine, provide numerous proteins that would be used as biomarkers to improve diagnosis.^72^ Global expression analysis of proteins is one of a kind states of the sickness represents an effective tool for a right away evaluation of signalling pathways and impaired capabilities of individual cells.^73^ Differentially expressed proteins in schizophrenia proteomic research were involved in the neuronal transmission, synaptic plasticity, and neurites outgrowth, consisting of numerous cytoskeletal parts.^74^ Relationships between schizophrenia and protein profiles exist in many brain areas, consisting of the frontal or prefrontal cortex, the anterior cingulate cortex, the corpus callosum, the temporal lobe neocortex, the hippocampus, and thalamus.^75^ The prefrontal cortex is the brain area most considerably studied utilising proteomics studies.^76^ Worldwide expression examination of proteins in one of a kind stages of the sickness presents a robust tool by directly assessing signalling pathways and impairment structures of person cells.^77^ However, the general studies using body fluids that as compared to brain tissue present some discrepancies and this lack of tissue biomarker detection can be instigated with the aid of low ranges of the release of disease-related proteins from tissues to body fluids.^78^

### Metabolic biomarkers

Genes, gene expression products (i.e., transcripts and proteins), and metabolites are the prominent biomarker families.^79^ The metabolome is defined as the set of metabolites present in a given biological system, fluid, cell, or tissue at a given time.^80^ The topological connections among these molecules define the groups, and the characteristic displays how the system evolves concerning metabolic fluxes and environmental stimuli.^81^ Numerous metabolomics studies confirmed an attempt to define higher the pathways altered in schizophrenia and its therapeutics.^82^ Previous metabolomics research on schizophrenia and associated psychoses have highlighted the importance of glucoregulatory tactics and tryptophan metabolism in psychosis. Lipidomics methods have recognised specific drug-reaction profiles for three commonly used strange antipsychotics (olanzapine, risperidone and aripiprazole).^83^ It is far recognised that schizophrenia is associated with increased peripheral total triglycerides and insulin resistance, but this metabolic abnormality has generally been attributed to antipsychotic drug precise side effects.^84^ Metabolomics can discover untimely biochemical modifications in disorder and lets in developing predictive biomarkers.^85^ In fact, metabolomics appears promising for analysing and identifying metabolic functions that describe certain pathological and physiological states.^86^ However, and metabolomics biomarkers need to be helpful for analysis, therapeutics and practical evaluation, the discovery of novel drugs and healing efficacy tracking.^87^

### Inflammatory markers

A high risk of inflammation can indicate the disease early, even though it is not specific to the disease. Inflammatory cytokines such as interleukin-1 beta and interleukin-17 are blood-based biomarkers that help predict the conversion of high-risk patients to ultra-high risk or complete psychosis.^88^ Interleukin-6, tumour necrosis factor, SIL-2R, and interleukin receptor antagonist are other peripheral markers significantly elevated in schizophrenia and bipolar disorder patients.^89^

### Biomarkers of oxidative stress

Reactive oxygen species (ROS) is a significant biomarker for oxidative stress, which results from abnormal levels of free radicals and superoxide residues in the plasma. It may damage the cell and results in impaired neurotransmission, which is pronounced in schizophrenia. RBC catalase, RBC superoxide dismutase, and plasma nitrite are elevated in patients with schizophrenia compared to healthy adults.^90^

### Miscellaneous

Other varieties of biomarkers will develop in the schizophrenia discipline. The evolution of techniques can offer new and exciting feasible biomarkers that do not have a finite boundary. In these studies, we located a few biomarkers that might be correctly correlated with schizophrenia. There may be promising biomarkers, like sleep deprivation, the endogenous salivary neuromodulator kynurenic acid degrees or the lipid profiles detected in peripheral pores and skin, including the ceramide.^91^ The latest attention points to biomarker discovery are encouraged by way of new molecular biology strategies that can find relevant markers fast and without a detailed evaluation of the mechanisms of the disease.^92^

## CNS drug delivery and function of the BBB

A significant factor determining the therapeutic efficiency is the bioavailability of the drug at the target site of action. In a neuropsychiatric disorder like schizophrenia, crossing the BBB is a significant concern during drug development. The BBB is a selective semipermeable separation that protects the brain from foreign pathogens. The BBB limits the movement of peptides from the apical membrane surface to the basolateral membrane surface (transcellular), also restricts the passage of substances through intercellular space between the cells (paracellular).^93^ This imposes a great challenge for drug transport across the BBB. The blood capillaries of the CNS are distinct compared to those of other organs present in the body due to the absence of tiny pores that are seen in other capillaries, which allow passage of some substances into respective organs.^94^ Astrocytes are star-shaped glial cells that form a solid envelop over the CNS and maintain homeostasis.^95^ They also function by transmitting nerve impulses and supplies nutrients to the neurons. Microglia act as inflammatory mediators in the CNS and act by destroying the invading pathogen by phagocytosis.^96^ There are other types of cells like pericytes involved in blood vessel formation, protection of BBB, and host immune response in the CNS.^97^ Endothelial cells constitute the brain’s blood vessels and hold together by tight junctions. These endothelial cells form a physical separation between brain and blood components. With the aid of carriers, enzymes and various drug transport mechanisms allow the transport of substances across the brain.^98^

### Drug transport across BBB

In order to gain more knowledge on drug transport across BBB, specific models have been developed like brain uptake index, which established transport of most of the substances across BBB, Co-culture technique, Transwell monoculture and In-silico models.^99^

#### Passive transport across BBB

The most simple and easy method of drug transport across the BBB is passive transport. It favours the passage of small, lipid-soluble molecules across the physiological barrier without the expenditure of energy. The degree of penetration of the molecule increases with the increased lipophilicity of the molecule. One factor determining the permeation of a molecule is the hydrogen bonding present within the molecule.^100^ Lesser the hydrogen bond, the more will be the permeation of the substance across the barrier. Drug classes like opioids and steroids favour passive diffusion for transport across BBB. Passive diffusion can be of two types, simple diffusion and facilitated diffusion. Simple diffusion allows the transport of hydrophilic molecules from one cell to another through intercellular spaces in BBB. Lipophilic molecules follow a transcellular pathway for transport.^101^ Facilitated diffusion is a type of passive diffusion in which carrier proteins facilitate the transport of drug molecules. The molecule to be transported (e.g. zidovudine) binds to the carrier protein, undergoes a conformational change that transports the molecule through a concentration gradient. After crossing the physiological barrier, the carrier detaches from the drug for exerting its action.^102^

#### Endocytosis

The process by which a substance is engulfed by the cell membrane forming a vesicle and transported to the other side of the barrier is termed endocytosis. Endocytosis is generally divided into three types, i.e. phagocytosis, pinocytosis and receptor-mediated endocytosis. Out of which receptor-mediated endocytosis is more critical in schizophrenia in which this mechanism transports hormones like insulin, enzymes and transport proteins. ^103^

Endocytosis, specifically clathrin-mediated endocytosis (CME), is involved in the pathophysiology of schizophrenia which is based on the evidence that dysbindin, a candidate gene for schizophrenia, was linked to CME.^104^ Dysfunction in the synapses, changes in the white matter and impaired neurodevelopment are affected by CME in schizophrenia. Any disturbance in the process of endocytosis can affect the brain’s normal functioning, including impaired neurotransmission. Antipsychotics employed in treating schizophrenia act by strongly inhibiting CME and thus effectively blocks the formation of coated pits.^105^

#### Active transport across BBB

Active transport is the mechanism by which the molecules are transported against a concentration gradient with energy expenditure. Certain antibiotics, anticancer agents, opioids are transported across BBB by active transport.^106^ Certain drug molecules make use of carrier molecules for easy transport across CNS. Active transport is of two types, influx and efflux transporter. Influx transporter facilitates the transport of hydrophilic molecules into the brain with the help of carrier proteins, and the drugs transported by this mechanism include methotrexate and digoxin.^107^ ABC transporters are a type of efflux transporter which transport the molecules out of the cell or inhibit the entry of the drug into the brain.^108^ Solid-lipid carriers (SLCs) are secondary active transporters involved in the pathophysiology of schizophrenia. SLCs are involved in the GABA-glutamine cycle. Its dysfunction results in excitotoxicity due to the accumulation of glutamate in the synapse and a reason for schizophrenia-like brain disorders.^109^

## Challenges faced during CNS drug delivery

Crossing the BBB is a significant challenge faced by drugs in the treatment of schizophrenia. The physiological barrier restricts the movement of drugs like antibiotics, anticancer drugs, analgesics. Certain factors create problems that hinder the transport of drugs across BBB. Therefore modification of drugs should be done concerning their physicochemical properties to easily cross BBB and exert its action on the target site. Several studies have been done so far, which showed the importance of their interpretation of in vitro models that cross the BBB. However, the primary concern in developing a novel therapeutic strategy for neuropsychiatric disorder must be to overcome the negative factors that challenge drug transport across BBB.^110^ These factors can be lipophilicity of the drug, size of the molecule, conformation of the molecule, and property of plasma binding of the molecule. In addition, the bioavailability of the drug in the CNS may be low even though a sufficient quantity is administered. The drug may bind to some plasma proteins and reduce the concentration of the drug available to CNS or undergo inactivation due to certain enzymes present in the circulation. Hence, these factors necessitate novel therapeutic agents, which may overcome the difficulty to cross the selective barrier. One therapeutic strategy that facilitates targeted drug delivery with sufficient bioavailability includes nanoformulation approaches described in later sections.

## Strategies for enhanced CNS drug delivery across BBB

**Figure 3:**
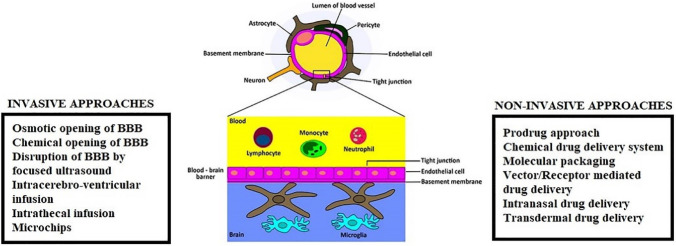
Diagrammatic representation of BBB and strategies for enhanced CNS drug delivery.

In order to overcome the obstacles faced during drug transport both at the drug level and at the BBB level, various approaches have been established to facilitate the transport of drug across BBB, such as physiological, neurological and pharmacological approaches.^111^ Neurological approaches mainly focus on bypassing the BBB and deliver the drug directly to the target site of action, thus avoid undesirable peripheral effects of the drug. This method found to be insecure and invasive observed to be less adopted compared to others. Physiological approaches include carrier-mediated or vector-mediated transport of drug that protects the drug from unfavourable effects and easy drug delivery across the BBB. Physiological approaches are a well-accepted and efficient method that facilitate drug transport using liposomes and other carriers.^112^ Figure 3 illustrates the diagrammatic representation of BBB and strategies for enhanced CNS drug delivery across BBB.

### Non-invasive chemical approaches

#### Prodrug approach

A prodrug is an inactive part of the drug which gets converted to its active form after metabolism. Novel therapeutic approaches also focus on the prodrug approach for a better clinical outcome. Metabotropic glutamate receptor is involved in the pathophysiology of schizophrenia, and their agonists are promising targets for the therapy. MGS0274 besylate is an ester prodrug that acts as a metabotropic glutamate receptor-2 agonists developed recently to treat schizophrenia.^113^ Predisposition in monkey and rat model indicates its safety in human models with an oral bioavailability 20-times more than active form. This prodrug can effectively cross the BBB due to its lipophilicity, and targeted drug delivery is established. Another study conducted by Mezler et al led to developing a selective metabotropic glutamate-2/3 receptor agonist LY-2140023, a methionine amide prodrug of LY-404039.^114^ This prodrug is in the early stage of clinical trials. In contrast, initial test results provide a ray of hope in the treatment of schizophrenia.

#### Chemical drug delivery system (CDDS)

Chemical drug delivery is a promising strategy to enhance drug transportation across BBB. Apart from the prodrug approach, CDDS need only an activation step.^115^ They can deliver the drugs at a controlled rate for a predetermined time without delay in the onset of action and bypassing first-pass metabolism. This novel drug delivery system depends upon the chemical reaction by which the drug gets released from the polymer which holds it.^116^ The polymer gets degraded within the body after use. The biodegradable polymers used for this purpose include polylactides, polyglycolide, polylactide-co-glycolides. CDDS can act by any three methods, either by enzymatic activation, or target site-specific enzymatic activation or by receptor-mediated CDDS.^117^ Dihydrotrigonelline is the common substrate to target the molecule, which is highly lipophilic and can deliver drugs into the CNS.^118^ Another example for this type of drug delivery is dihydropyridine which can cross the BBB and exerts its action with sufficient bioavailability.^119^

#### Molecular packaging

Molecular packaging is a technique to improve the penetration of the antipsychotic drug through BBB. The primary goals achieved by molecular packaging include enhanced passive transport by increasing the lipophilicity of the molecule, increasing the enzyme stability, thereby preventing immature degradation of the molecule and targeted drug delivery by blocking the lock-in mechanism.^120^ In this technique, a bulky group with a peptide fragment forms the core, protected by certain groups from peptidases’ action and enhances BBB penetration. There is available evidence of a new target in the treatment of schizophrenia, the GABAA receptor. These are targets for benzodiazepine action with sedative and hypnotic activity. Thyrotropin-releasing hormone and benzodiazepines are drugs investigated for establishing a molecular packaging approach, and other results have to be obtained from trials.^121^

### Non-invasive biological approaches

#### Receptor-/vector-mediated drug delivery

Receptor or vector-mediated drug delivery is done by forming a complex between a non-transportable molecule with a transportable agent such as a vector that carries the molecule across BBB through receptor-mediated transcytosis. The process of endocytosis can be enhanced by binding the vector on the receptors present in the luminal surface of the endothelial cells.^122^ The bond formed between the vector and the drug molecule gets cleaved when it crosses the BBB, and the pharmacologically active moiety is released for its action. Transferosomes is found to be the carrier of asenapine, increasing BBB penetration in in-vitro and in-vivo models is an example for receptor-/vector-mediated drug delivery.^123^

#### Viral vector-mediated drug delivery

Viral vector-based gene therapy is a novel and promising drug delivery approach adopted in many diseases, including autoimmune disorders and cancer, directly injected into the cerebral ventricles. The virus infects the cells and releases the drug by enclosing it in vesicles. The vesicles being lipophilic carries the drug into the CNS and release the drug after crossing BBB. The viruses mainly used for this purpose include lentivirus, retrovirus, adeno-associated virus, and helper-dependent adenovirus.^124^

### Alternative approaches

#### Intranasal drug delivery

**Figure 4:**
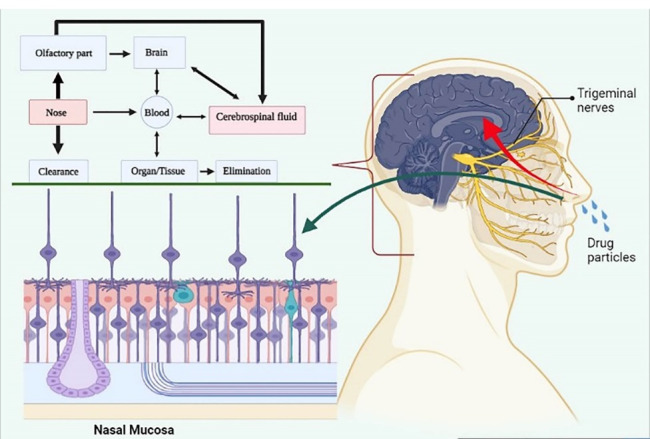
Diagrammatic illustration of intranasal drug delivery.

Intranasal drug delivery is a widely recognised approach that bypasses the BBB and delivers the drug to the CNS through olfactory receptors and Figure 4 shows diagrammatic illustration of intranasal drug delivery.

Antipsychotics employed in the treatment of schizophrenia reduces to as much as by three quarters when administered via oral/parenteral route.^125^ In addition, the drug’s metabolism before reaching the CNS results in peripheral side-effects including weight gain, diabetes, drug-induced locomotory difficulties.^126^ The advantages of intranasal drug delivery include high bioavailability, lower side-effects, self-administration, rapid onset of action, controlled release of the drug. Some of the disadvantages of this method include nasal irritation, local adverse effects, and mucociliary clearance. In an intranasal drug delivery study conducted by McMaster University, Canada, a nasal spray was engineered for delivering the schizophrenia medications into the CNS directly, bypassing the BBB.^127^ In this study, the drug directly enters the brain through the olfactory nerve. The drug developed was bonded with corn starch nanoparticles; spraying along with a natural polymer obtained from crabs can penetrate the nasal epithelium, facilitate slow release of the drug, and remains in circulation for about three days. This concept was developed in rat models and demonstrated advantages like reducing dosing frequency, controlled drug delivery, enhanced bioavailability. Patel and colleagues’ nose-to-brain drug delivery study show promising results in treating schizophrenia using olanzapine loaded microemulsions; in the preparation, oleic acid was used as the oil phase, Kolliphor® RH40 as the surfactant and Transcutol as co-surfactant.^128^ A mucoadhesive polymer, polycarbophil, was used to enhance the adhesion to the nasal mucosa. A pharmacodynamic study was conducted in mice using apomorphine as a dopamine agonist model. It showed better therapeutic outcome.^129^ Intranasal drug delivery is effective and safe in patients with schizophrenia and improves their quality of life.

#### Intracerebroventricular drug delivery and cerebral implants

Intracerebroventricular drug delivery is when the drug is administered directly into the cerebrospinal fluid and exerts targeted action. A study conducted by Esen-Sehir et al using clozapine in mice model showed improved open field activity and prepulse inhibition in female mice and not in male mice.^130^ The drug injected Intracerebroventricular route did not show neurological or behavioural deficit in mice. This route of drug administration has advantages like bioavailability, longer half-life, reduced peripheral side-effects, bypassing first-pass metabolism.

#### Transdermal drug delivery

Transdermal administration of drugs is an emerging option for most drugs, having advantages like low cost, self-administration, rapid onset of action. For drugs having a short half-life, transdermal administration of drugs reduces variations in plasma concentration. Clozapine is a second-generation antipsychotic drug dissolved in a lipophilic phase like oleic acid using Transcutol and tween 20 as surfactants that form o/w nanoemulsion with enhanced transcutaneous absorption.^131^ Another trial shows the application of proniosomes of risperidone with high entrapment efficiency. It uses spans over tweens which results in an increased drug release profile. Spans are lipophilic, which forms a lipid bilayer into which lipophilic risperidone is entrapped. The drug entrapped between uniform bilayer prevents the leakage of the entrapped drug. The absorption is facilitated initially by releasing risperidone from the vesicle, and later the drug is diffused into the skin through swollen bilayers of niosomes.^132^ Transdermal drug deliveryshows improved bioavailability of about 92% and increases the half-life to 8h compared to conventional methods.^133^ The only transdermal drug approved for the treatment of schizophrenia is Asenapine, approved in 2019.^134^ The results from two doses of transdermal Asenapine was found to be superior to the control group. However, the release date of the drug is yet to be announced.

#### Iontophoretic drug delivery

Iontophoresis is a novel method used to apply drugs in the form of ions by passing electric current. Some iontophoretic devices deliver the drugs into the CNS through the olfactory route. In-vitro iontophoretic transdermal drug delivery has also been investigated in pig models. An example of this is the administration of haloperidol; an antipsychotic used to provide symptomatic relief in schizophrenia.^135^ The technique showed improved drug penetration due to the ionic strength and current density, and the penetration was not affected by drug concentration.^136^ This proves to be a promising method in drug delivery for those having poor penetration into the brain, but further research has to be done.

## Nanoformulation approaches in schizophrenia

**Figure 5:**
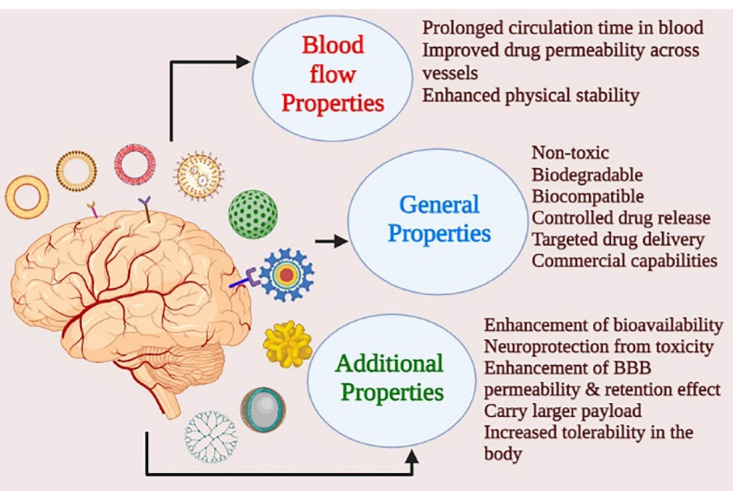
Advantage of nanoformulations over conventional approach.

Nanoformulations and nanotechnology approaches are milestones in medicine and pharmaceutical science that address several patient-related and formulation-related barriers such as bioavailability, tissue penetration, receptor binding affinity, controlled drug release, targeted drug delivery, and reducing peripheral side-effect. Nanoformulations are now the treatment choice for many diseases, including schizophrenia, other than autoimmune disorders and cancer. Nanomedicine is a better platform for novel therapeutic approaches that prove outstanding for targeted drug delivery and minimise adverse effects.^137^ The therapeutic outcome is dependent on the physicochemical properties of the drug, including its lipophilicity, molecular weight, charge of the molecule or ion, solubility. These factors are modified in the nanoformulation to achieve a better clinical outcome. Currently, in schizophrenia, nanoformulations are considered to be the preference over any other treatment strategies and Figure 5 shows the advantage of nanotherapeutic approaches over the conventional ones.

In addition, nanoformulations have advantages like favouring administration through all possible routes such as oral, parenteral, nasal and other routes. Nanoparticles are the functional unit in this method that facilitates effortless drug delivery across BBB, site-specific action, and biodegradable. The nanoparticle-based drug delivery shows promising results in cancer chemotherapy. The primary nanoparticles used in the treatment of schizophrenia include polymeric nanoparticles (PNPs), solid lipid nanoparticles (SLNs), liposomes, microemulsions, dendrimers, micelles, and hydrogels.^138^

### Therapeutic nanomaterials in clinical practice

Nanotechnology is a broad term that describes various devices, carriers, and materials that aid in drug transport and therapeutic action. Nanotechnology can be divided into two major types, nanomaterials and nanodevices. Nanomaterials act as nano-sized lipophilic carriers that help in drug transport across BBB. They can be again further classified according to chemical property as nanostructured and nanocrystalline particles. Nanostructured particles are microstructured particles whose size is typically in the range between 1-10 nm. They can be of three types, polymer-based, non-polymer based and lipid-based nanostructured materials. Polymer-based nanoparticles include PNPs, dendrimers, micelles, protein nanoparticles, nanogels and drug conjugates. Lipid-based nanoparticles include liposomes and SLNs. Polymer-based and SLNs have wide application in the clinical sector. Non-polymer based nanoparticles include carbon nanotube, metallic nanoparticles, Nanodiamond, quantum dots and silica-based nanoparticles. They are mainly intended for photodynamic applications and in the diagnosis of disease based on their structure.^139^ Nanocrystalline are materials with single or multiple phase polycrystals with an order of 1-10 nm. These nanomaterials have their own merits and demerits, which still challenge nanomedicine from being a standard therapeutic approach.^140^ Novel nanosized drug delivery systems and some of the examples have been enlisted in Table 1.

**Table 1:** Classification of therapeutic nanocarriers in clinical practice

**A. Nano structured**	
Polymer based	Dendrimers, Polymeric nanoparticles, Micelles, Drug conjugates, Protein nanoparticles, Nanogels
Lipid based	Liposomes, Exosomes, Solid-lipid nanoparticles
Non-polymeric	Carbon nanotubes, Nanorods, Metallic nanoparticles, Quantum dots, Silica-based nanoparticles
**B. Nanocrystalline**	Nanocrystals conjugated with ligands, stimuli-responsive polymers, encapsulation, cross linking

#### Polymeric nanoparticles

*PNP*, composed of natural or synthetic polymers is a promising approach for CNS drug delivery due to their non-toxicity, biocompatibility, biodegradable nature and non-immunogenicity.^141^ To lower the adverse effects and high immunogenicity of synthetic polymers, such as polylactic acid, polyester forms can be used. Natural polymers like chitosan and gelatin are more effective than synthetic polymers because of their less immunogenicity and adverse effects.^142^ PNPs composed of a matrix system in which it is dispersed uniformly. Based on the composition, it can be nanospheres. The active drug moiety is directly dispersed into the matrix, or it can be nanocapsules in which the therapeutic moiety is enclosed in the polymer matrix.^143^ In a recent study conducted by Rukmangathen et al in which antipsychotic drug Risperidone is formulated within chitosan nanoparticle and is evaluated for intranasal bioavailability in the treatment of schizophrenia. The result was astonishing that risperidone-loaded chitosan nanoparticle showed an entrapment efficiency of 78%, and bioavailability was found to be significantly higher than conventional approaches.^144^ The advantage of PNPs is that the surface can be easily modified and functionalised so that the specificity of the particle can be enhanced. In a study conducted by Panda et al, the current antipsychotic drugs are primarily given for schizophrenia, risperidone and clozapine, entrapped in PNPs polylactide-co-glycolide and is formulated using spray drying technique.^145^ Adequate and appropriate drug release from the spray can be attained using polymers of different molecular weights. Low molecular weight polymer has more efficiency in entrapping the drug. There was no significant interaction between the drugs observed. Now exciting research done by Ranka and colleagues is that they prepared a copolymer system with conjugated polymeric silica nanoparticles and is experimented with to provide stability to O/W emulsion. Emulsion gets released into the systemic circulation only at elevated temperature conditions. Those mentioned above attain the stability of preparation, and systemic effect is produced only at the desired site at a predetermined time.^146^ But formation and aggregation of toxic monomers and toxic degradation of the nanoparticles may lead to induction of ROS, which is a drawback of this system.

#### Gold nanoparticles

Inorganic nanoparticles and compounds of inorganic nanoparticles with natural materials form combinational products with physical, chemical, optical, and electrical properties, making them extraordinary and more relevant than other nano-size materials. Nanoparticles are promising multi-purpose materials since they can be utilised for imaging and therapeutic abilities. Gold nanoparticles explicitly have been effectively related to some antipsychotic medication. Gold nanoparticles are extensively used in chemotherapeutics, while their role in neuropsychiatric disorders is in prime role to be considered. Apart from that, certain antipsychotic medicines can be prepared in a type of nanosystem with the help of gold nanoparticles, which may improve their bioavailability. Thioridazine, which has a place with phenothiazine regular antipsychotic class of medications, in some cases utilised for the treatment of schizophrenia, is one such drug.^147^ It has been indicated that utilising gold nanoparticles is conceivable to produce ethyl cellulose microcapsules containing thioridazine. Nanoparticles significantly developed the thioridazine epitome, which might be helpful for additional psychopharmacology investigation. Even though surface modification of gold nanoparticles reduces the toxicity, the toxicokinetic profile of gold nanoparticle is a challenge to be critically considered.^148^

#### Liposomes

Liposomes are microscopical vesicular structure with phospholipid bilayer enclosing in an aqueous layer. Liposomes are amphiphilic with a polar head and hydrophobic tails. Liposomes have versatility in therapeutic applications in immunology, tumour therapy, gene therapy, antiviral therapy, and the most important is drug delivery.^149^ Liposomes are composed of biodegradable and biocompatible material, which make them safe for drug delivery. Since liposomes are composed of phosphates, they can easily fuse with a cell’s plasma membrane, which is more efficient than pinocytosis. Thus, liposomes have been proposed for utilisation for their application in research, industry, and medicine, especially for the utilisation as transporters of symptomatic and therapeutic drug molecules. The unique ability of liposomes to entrap drugs in both an aqueous and a lipid phase makes such delivery systems attractive for hydrophobic and hydrophilic drugs, and such encapsulation proved to reduce drug toxicity while retaining or improving the therapeutic adequacy.^150^ Liposomes are the most commonly used carrier of formulations for brain delivery in vivo and proved to enhance the bioavailability of numerous deprived agents into the brain, primarily in people with schizophrenia. Quetiapine fumarate is an antipsychotic drug listed among poorly bioavailable drugs, formulated into nano-liposomes by Upadhyay et al, which showed improved bioavailability on administration with the nasal route. It concluded his results as a promising nanotherapeutic approach.^151^ Another atypical antipsychotic drug, amisulpride, used in the management of schizophrenia, is a drug with poor permeability, results in poor bioavailability. Since liposomes are the best choice for poorly soluble drugs, it has been loaded with cyclodextrin-liposomes to form amisulpride-cyclodextrin liposomes (AMS-CD) by Zeeshan et al. High entrapment efficiency and bioavailability for more than 2-folds was observed compared to conventional approaches.^152^ The major limitation faced by liposome includes the leakage of drug from encapsulation in some instances; some phospholipids may also undergo undesirable reactions like hydrolysis and oxidation. Further research must be done to overcome these limitations for a better formulation. Other than being expensive in the manufacturing process, the high cost of the raw materials used in liposomes is also limited to using them as carriers for the drug delivery system.^153^

#### Solid-lipid nanoparticles

SLNs are excellent carrier system composed of nano-sized lipid particles. The advantages of SLN include small size, large surface area, and high entrapment efficiency and thus improves bioavailability. SLNs are composed of a hydrophilic colloidal dispersion with the solid lipid matrix. The framework is stable at any physical conditions, and this contributes to its more comprehensive applications.^154^ SLN has a low drug loading efficiency reported in certain situations due to their perfect crystalline structure. Quetiapine fumarate is an antipsychotic ester drug used in the management of schizophrenia. The drug was loaded with SLN prepared by microemulsion technique by Jian et al and was evaluated in rat models via nose-brain administration. QF-SLN-gel was found to be physically stable, and an excellent brain delivery model was demonstrated.^155^ Another investigation by Emil et al was by formulating the glyceryl monostearate nanoparticles with olanzapine. The SLNs loaded with olanzapine was further coated with polysorbate 80, and receptor-mediated endocytosis was enhanced.^156^ The nano-sized particles (SLN) show high entrapment efficiency with minimum side effects and prove to be promising approaches in managing schizophrenia.

#### Dendrimers

Dendrimers are nano-sized, branched, highly ordered, homogenous, a monodispersed structure consisting of a central core, an inner and an outer shell. These branches can be modified, which helps us to fabricate their size in nanoscale. They can be modified into spheres where they form cavities, and the drug can be entrapped.^157^ Additional biocompatible compounds can be added to the free ends of dendrimers, where they render the molecule to be less toxic and less immunogenic. Such modifications also entertain high tissue permeability and target-specific drug delivery. Dendrimers can be encapsulated or complexed to utilise as carriers for pharmacologically active substances like vaccines, antisera, medications or genes. Polyethene imine and chitin are some of the polymers used in clinical practice nowadays. Haloperidol is a poorly aqueous soluble drug made into a dendrimer-based formulation targeted to CNS by intraperitoneal (via peritoneal cavity) and intranasal route in an evaluation done by Katare et al. The results were promising that the bioavailability of the drug in CNS was increased and resulted in the high distribution of haloperidol in brain tissues.^158^ The solubility of the drug in the aqueous solution was found to be increased by the formulation for more than 100-fold. This study indicates that the dendrimer formulations improve the absorption of water-soluble drugs in the CNS to a great extent. Nevertheless, the difficulty synthesising many dendrimers for a clinical trial is a challenge for its efficacy results.^159^

#### Micelles

Micelles are polymers intended for the systemic administration of hydrophilic drugs. They form aggregates in solution, and the size is in a range less than 100 nm. The arrangement of component molecules in the polymeric micelles is similar to a sphere in which the active moiety is either dispersed throughout or inside the polymer matrix like the hydrophobic part is surrounded by a hydrophilic part. The hydrophilic part protects the system from the uptake by the reticuloendothelial system and ensures high stability to the system. In addition, the hydrophobic part entraps the organic, hydrophobic drug moiety. They show a prominent drug delivery approach, targeted drug delivery with appropriate safety and efficacy.^160^ A micellar liquid chromatographic technique done on antipsychotic clozapine in vivo is a suggested diagnostic tool for intoxication with other drugs in the emergency management of schizophrenia.^161^ Improved permeability, dissolution and bioavailability of micelles loaded with aripiprazole is evaluated in vitro and in vivo by Piazzini et al in the management of schizophrenia.^162^ Another study conducted by Singla et al reveals that sustained release of drug action is observed on solubilising the hydrophobic drugs clozapine and oxcarbazepine in different molecular weight pluronic mixed micelles in vitro and in vivo.^163^ This method is promising for drugs having poor solubility and penetration across BBB.

#### Nanogels

Nanogels are a novel therapeutic approach used as a drug delivery system in Alzheimer’s disease, schizophrenia, migraine and depression. The intranasal route of nanogel administration through the olfactory nerve effectively manages CNS disorders with an astonishing neuroprotective effect. Nanogels are hydrogel on a nanoscale. They are prepared from natural, semi-synthetic and synthetic polymers and can occur in various shapes and structures with cross-linking over the polymers. The polymer swells and increases drug loading efficiency, and the cross-linking provide high stability to the structure.^164^ They can pass through BBB and get released at the target site of action. Khan et al developed a nanogel using a polymer poloxamer-407 cross-linked with 2-acrylamide-2-methyl propane sulfonic acid by increasing the solubility in tissue fluids’ help cross-linking agent, methylene bisacrylamide. The nanogels formulation evaluated in vitro for its bioavailability and toxicity studies done on rabbits gives satisfying results.^165^ The bioavailability of the drug was found to be increased by about 38-folds. In addition, low toxicity, increased stability, solubility, and improved safety and efficacy provide a new insight for developing newer antipsychotic nanogel formulations.

#### Microemulsions and nanoemulsions

Emulsions are dispersions made up of two immiscible liquid phases mixed using mechanical shear, and a surfactant is used. They solubilise hydrophobic or oil-soluble drugs, enhance drug absorption, improve topical absorption of drugs, mask the disagreeable taste and odour of drugs, and magnify the palatability of nutrient oils.^166^ In advance, they increase the bioavailability of the lipophilic drug, which is our concern. Aripiprazole is considered a third-generation antipsychotic drug with excellent therapeutic efficacy in controlling schizophrenia symptoms and was the first atypical antipsychotic agent to be approved by the US Food and Drug Administration.^167^ Formulation of nanoemulsion-containing aripiprazole was carried out using high shear and high-pressure homogenisers. A mixture experimental design was selected to optimise the composition of nanoemulsion. The petite droplet size of an emulsion can provide an effective encapsulation for the delivery system in the body. The effects of palm kernel oil ester, lecithin, Tween 80, glycerol, and water on the droplet size of aripiprazole nanoemulsions were investigated.^168^ The mathematical model showed that the optimum formulation for preparation of aripiprazole nanoemulsion having the desirable criteria was 3.00% of palm kernel oil ester, 2.00% of lecithin, 1.00% of Tween 80, 2.25% of glycerol, and 91.75% of water. Under optimum formulation, the corresponding predicted response value for droplet size was 64.24 nm, which showed an excellent agreement with the actual value (62.23 nm) with residual standard error < 3.2%.

## Discussion

Nanotechnology-based drug delivery system help in targeted drug delivery with reduced peripheral side effects. It also offers specificity, increased lipophilicity, improved membrane permeability, improved patient compliance, and reduced treatment cost. In major CNS diseases like schizophrenia, lack of specific treatment leads to undesirable effects in the patient. Novel drug delivery system using PNPs, nanogels, nanoemulsions aids in improved drug delivery to the target action. Various hypothesis other than focussing on dopamine could improve the treatment approach since antidopaminergic agents could relieve only antipsychotic episodes. In addition, nanodevices are also structured to diagnose and deliver the drug molecules with minimum invasion. Fluid biomarkers are a reliable method for the early diagnosis of the disease.

Many drugs have been introduced in the past, but most of them remain unsafe for use. Recent studies focus on specific other categories of drugs such as acetylcholinesterase inhibitors like donepezil that appears to enhance cognitive functioning in patients with schizophrenia. A partial dopamine agonist like aripiprazole also appears to balance out the activity levels between the presynaptic and postsynaptic dopamine receptors. In addition, the therapeutic implications of the NMDA receptors and Cox-2 inhibitor as adjunctive treatment for schizophrenia are under investigation. Certain drugs such as asenapine reported poor bioavailability as a psychopharmacological agent in the past, now has been loaded with nanostructure lipid carriers to enhance the uptake via the intranasal route. It is now possible to map candidate regions more comprehensively with the new SNP (single nuclear polymorphism) information to better localise linkage regions. Schultze-Lutter and Klosterkötter^169^ have developed specific instruments for the prediction of schizophrenia. All point towards the new advancements in the treatment strategies. New strategies, like three potential treatments—ITI-007 (Intra-Cellular Therapies), MIN-101 (Minerva Neurosciences), and NaBen (SyneuRx)—are aimed at managing the negative symptoms of the disease. Finally, three formulations of risperidone—risperidone implant (Braeburn Pharmaceuticals), risperidone ISM (Rovi), and RBP-7000 (Indivior)—are expected to offer improved safety profiles in the treatment of schizophrenia.

Neuroimaging techniques can analyse the brain structure for identifying the abnormal. Genetic and epigenetic biomarkers could identify the hidden genetic predisposition underlying schizophrenia. Novel approaches can act on various stages of the disease like diagnosis, treatment, and prognosis will improve the patient’s quality of life without imposing a burden on the patient in the name of treatment.

## Conclusion and future perspectives

Schizophrenia is a highly heritable, chronic, severe neurodevelopmental brain disorder with a heterogeneous genetic and neurobiological background. A high emphasis is needed on transporting the drug across the physiological barrier, the BBB and the mode of transport of the drugs for a better therapeutic outcome. Fluid biomarkers have created an excellent scope for diagnosing the disease. With the help of these markers, identification of people with a risk of developing schizophrenia was made easy. There is a need to identify new biomarkers that are definite and stable and quickly identify the chances of getting the disease. In order to address the therapeutic interventions, an analysis of additional hypothesis is required. Based on the information available, the current treatment strategy mainly focuses on the dopaminergic hypothesis, which includes Dopaminergic receptor inhibitors and modulators, Glutamate hypothesis, 5HT-6 receptor hypothesis. The development of nanoscience created a way to the treatment of schizophrenia also. Nanomedicine approaches include liposomes, PNPs, dendrimers, nanoemulsions, nanocapsules and nanospheres, which forms an excellent approach because of its efficiency, specificity, low toxicity, ability to cross BBB and enhanced therapeutic effect.

In summary, several products are being developed to address the significant unmet needs in the schizophrenia marketplace. From the related articles and news, it is evident that schizophrenia is not still an interrogation; instead, it is on its way to being a solution for all the problems that would enhance the quality of life. In this review, we tried to address the significant challenges faced in the therapy of schizophrenia and these fluid biomarkers aid in the early detection of disease and the merits and demerits of various nanotherapeutic approaches adopted for enhanced CNS drug delivery in the management of schizophrenia.

## Ethical Issues

Not applicable.

## Conflicts of Interests

There is no conflict of interest.
